# Alleviating the toxicity concerns of antibacterial cinnamon‐polycaprolactone biomaterials for healthcare‐related biomedical applications

**DOI:** 10.1002/mco2.71

**Published:** 2021-05-18

**Authors:** Jubair Ahmed, Merve Gultekinoglu, Cem Bayram, Didem Kart, Kezban Ulubayram, Mohan Edirisinghe

**Affiliations:** ^1^ Department of Mechanical Engineering University College London London UK; ^2^ Department of Basic Pharmaceutical Sciences Faculty of Pharmacy Hacettepe University Ankara Turkey; ^3^ Department of Nanotechnology & Nanomedicine Division Institute for Graduate Studies in Science & Engineering Hacettepe University Ankara Turkey; ^4^ Department of Pharmaceutical Microbiology Faculty of Pharmacy Hacettepe University Ankara Turkey

**Keywords:** antibacterial, biomaterial, cinnamon, cytotoxicity, fiber

## Abstract

Fibrous constructs with incorporated cinnamon‐extract have previously been shown to have potent antifungal abilities. The question remains to whether these constructs are useful in the prevention of bacterial infections in fiber form and what the antimicrobial effects means in terms of toxicity to the native physiological cells. In this work, cinnamon extract containing poly (ε‐caprolactone) (PCL) fibers were successfully manufactured by pressurized gyration and had an average size of ∼2 μm. Cinnamon extract containing PCL fibers were tested against *Escherichia coli, Staphylococcus aureus, Methicillin resistant staphylococcus aureus*, and *Enterococcus faecalis* bacterial species to assess their antibacterial capacity; it was found that these fibers were able to reduce viable cell numbers of the bacterial species up to two orders of magnitude lower than the control group. The results of the antibacterial tests were assessed by scanning electron microscopy (SEM). The constructs were also tested under indirect MTT tests where they showed little to no toxicity, similar to the control groups. Additionally, cell viability fluorescent imaging displayed no significant toxicity issues with the fibers, even at their highest tested concentration. Here we present a viable method for the production the non‐toxic and naturally abundant cinnamon extracted fibers for numerous biomedical applications.

## Graphical abstract

Cinnamon directly extracted into a polycaprolactone polymer base was spun with pressurized gyration to produce a high yield of cinnamon‐extracted bandage‐like fibers. These fibers where then characterized and tested for cytotoxity and antibacterial activity against *Escherichia coli*, *Staphylococcus aureus*, *Methicillin Resistant Staphylococcus aureus* and *Enterococcus faecalis*.

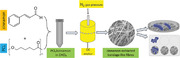

## 1 INTRODUCTION

In open wound settings, bacterial infections are especially dangerous due to the high number of infections seen in hospitals, which provide opportunities for multidrug resistant strains.^1^, ^2^, ^3^ Open wounds offer an ideal environment for pathogens (bacteria, fungi, and viruses), which are then free to invade the compromised defensive barrier, the skin. In order to overcome this problem, several approaches have been tried, these include antibiotics, silver (Ag) nanoparticles, zinc oxide and chlorhexidine.^4^, ^5^, ^6^ However, these antibacterial agents are limited by their toxicity and inability to combat antimicrobial resistance, which has led to an alarming increase in ineffective agents. Even with the wide usage of Ag nanoparticles as antibacterial agents, there remain reports on its cytotoxicity and genotoxicity.^7^ Recent studies have therefore focused efforts on alternative antibacterial approaches such as plant extracts, essential oils, or single chemical compounds as an alternative to antibiotics or synthetic antibacterial agents for wound management.^8^

Cinnamon is a popular spice that has a diverse primary use in culinary applications but has also been used as traditional herbal medicine in many cultures around the globe for centuries.^9^, ^10^ Cinnamon has long been utilized in an extensive range of applications without any notable side effects, for example in biblical times, cinnamon was used in rubbing oils as perfumes.^11^ Cinnamon acts as a natural coagulant and can prevent bleeding.^12^ Many reports into the medicinal uses of cinnamon see its utilization in treatment of inflammatory disorders, diabetes, neurological disorders, some cancers, and even cardiovascular diseases.^13^, ^14^, ^15^, ^16^, ^17^, ^18^, ^19^

Naturally occurring cinnamon belongs to the genus *Cinnamomum*, two main species exist *C. Cassia* and *C. Ceylon*.^20^ Although less treasured in terms commercial value due to its abundance in nature, *C. Cassia* differs from *C. Ceylon* in its flavor, it has a stronger flavor owing to its higher content of its main chemical components. Extractions of cinnamon contain a wide variety of compounds. The principle compound is thought to be cinnamaldehyde, but the spice also contains cinnamic acid and many other essential oils.^21^ Cinnamaldehyde is an oily liquid characterized by its yellow color and strong odor, it gives the cinnamon its spicy taste and has been used widely in cosmetics, as flavoring agents, and in perfume.^22^, ^23^, ^24^ Cinnamaldehyde can also actively inhibit growth of bacteria, yeast, and fungi.^25^, ^26^, ^27^, ^28^, ^29^ The antimicrobial action of cinnamaldehyde is instigated by its inhibition of ATPase activity, biosynthesis of the cell wall and its ability to degrade the structural integrity of microbial membranes.^30^, ^31^, ^32^ *C. Ceylon* contains a higher ratio of cinnamaldehyde than other species of cinnamon. Cinnamic acid is a white crystalline compound, which sees uses in flavorings and is a precursor in the production of the common sweetener, aspartame.^33^ Cinnamon also contains many essential oils, namely *trans*‐cinnamaldehyde, *α*‐cubebene, caryophyllene oxide, *L*‐borneol, *α*‐thujene, eugenol, *β*‐caryophyllene, *α*‐terpineol, caryophyllene oxide, L‐bornyl acetate, terpinolene, *E*‐nerolidol, and cinnamyl acetate.^13^, ^34^ *C. Cassia* contains considerably more coumarin, approximately three orders of magnitude higher. Coumarin is known to be toxic in nature and is a chemical defense in some plants.^35^ For these reasons, the toxicity of using cinnamon extracts are questioned and are thus explored in this work.

Previously, cinnamon‐extracted fibers were produced into poly (ε‐caprolactone) (PCL) bandage‐like fibers in three differing concentrations: 250, 375, and 500 mg/mL (C1, C2, and C3, respectively). These fibers were intended to function as wound healing constructs and were shown to be highly effective against the fungal species *Candida albicans (C. albicans)*.^36^ *C. albicans* can form biofilms in open wounds that harbor pathogenic and opportunistic bacteria, can lead to infections causing candidiasis and can also lead to some patients developing zygomycosis over time.^37^, ^38^ The antibacterial effect and cell study of cinnamon in this bandage form has not yet been tested.

PCL is a frequently used polymer in biomaterials, tissue engineering, and controlled delivery applications owing to its biocompatibility and biodegradability.^39^ PCL is a semi‐crystalline polymer with a low degradation rate and good mechanical properties allowing it to have a high degree of flexibility and tensile strength, making it suitable for use in healthcare applications.^40^, ^41^ PCL is a hydrophobic polymer, which is advantageous for the preparation of hydrophobic active ingredients in common organic solvent systems. Additionally, it has great compatibility with volatile solvents simplifying the production process.^42^

Given the beneficial nature of cinnamon, there remains a concern in the toxicity to human cells from such a potent antimicrobial agent. In this work, the antibacterial activity against *Escherichia coli (E. coli)*, *Staphylococcus aureus (S. aureus)*, methicillin resistant *Staphylococcus aureus (MRSA)*, and *Enterococcus faecalis* (*E. faecalis*) was determined for the cinnamon‐extracted bandage‐like fibrous constructs and their toxicity on cells. The use of natural materials can alleviate many of the long‐term side effects and concerns that may be had from using synthetic chemical compounds. Furthermore, naturally acquired materials are entirely biodegradable and can reduce the environmental impact caused by the pursual of antibacterial agents. In this work, PCL is used to produce four different sets of bandage‐like fibers. Cinnamon is then extracted using chloroform at three concentrations (C1, C2, and C3) and subsequently all polymeric fibers are created with 15% (w/v) PCL in chloroform, only differing in cinnamon concentration for equal comparison. The work carried out here shows how viable biomedical products can successfully and safely be created using abundant natural materials with modern materials engineering and manufacturing methods.

## 2 RESULTS

### 2.1 Structure and morphology of the fibrous constructs

**FIGURE 1:**
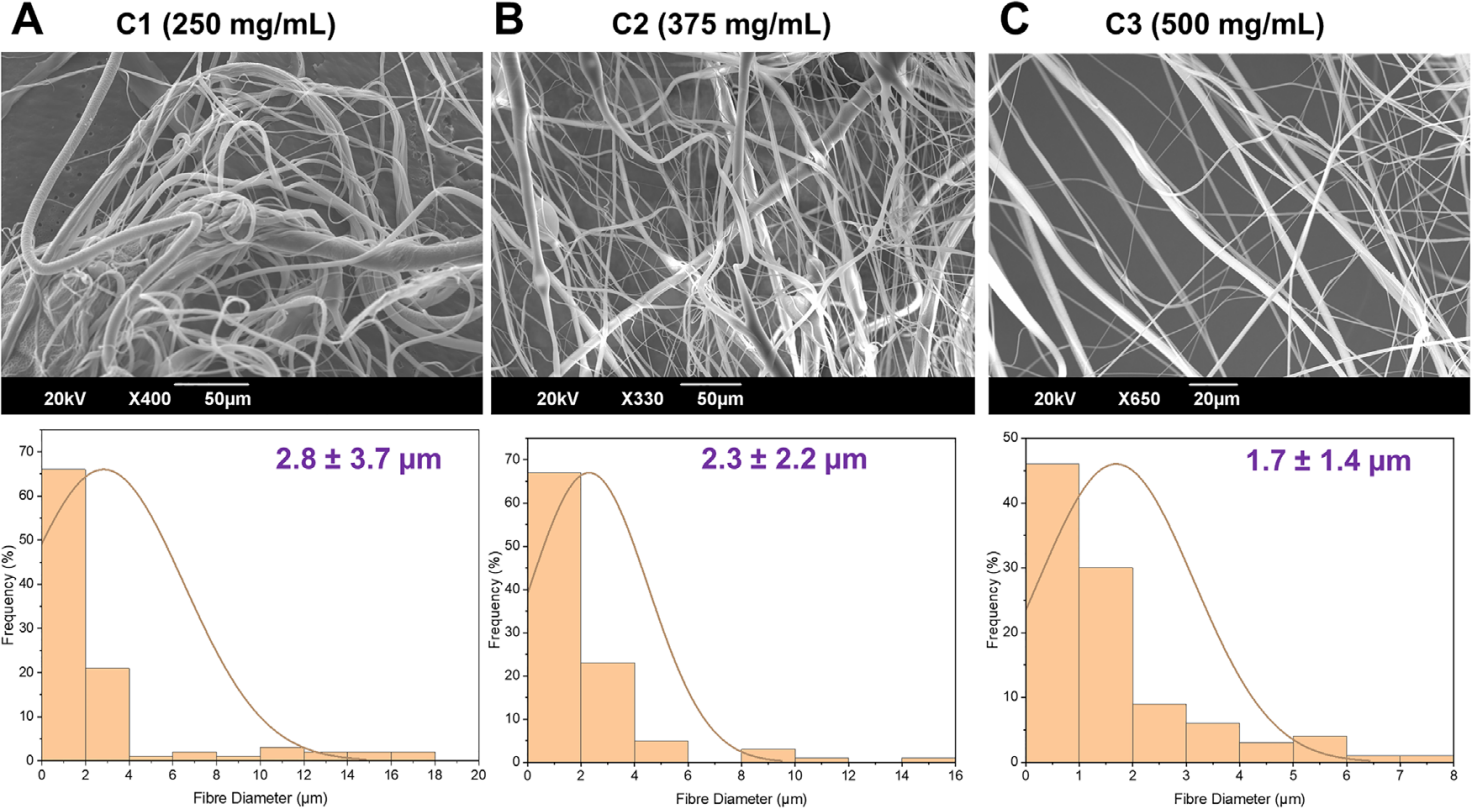
SEM micrographs of (A) C1, (B) C2, (C) C3 cinnamon‐extract containing fibers and the corresponding diameter distribution for these fibrous constructs (*n* = 100), C1: 15% PCL+250 mg/mL cinnamon, C2: 15% PCL+375 mg/mL cinnamon, C3: 15% PCL+500 mg/mL cinnamon

The produced fibrous constructs where formed into a mesh straight from the gyration vessel, these meshes resemble large bandages that can be cut, sterilized, and dressed directly onto several biomaterial applications for antibacterial purposes (surgical mesh, wound dressing, bandages, etc). Figure 1 shows the scanning electron microscopy (SEM) images and the fiber diameter distributions of the fibrous samples. The structure and the morphology of polymeric bandage‐like or scaffold materials can determine the bioactivity and the cellular interactions that can occur and are therefore subsequently important for a good wound healing environment.^43^ A smaller fiber diameter gives rise to a higher surface area to volume ratio, which further encourages fibroblast and epithelial cell attachment and proliferation. C1, C2, and C3 fibers only differed in the addition of cinnamon with increasing concentrations, which are denoted in Figure 1; all fiber samples in this work consisted of a base concentration of 15% (w/v) PCL.

### 2.2 Thermal gravimetric analysis of the fibrous constructs

**FIGURE 2:**
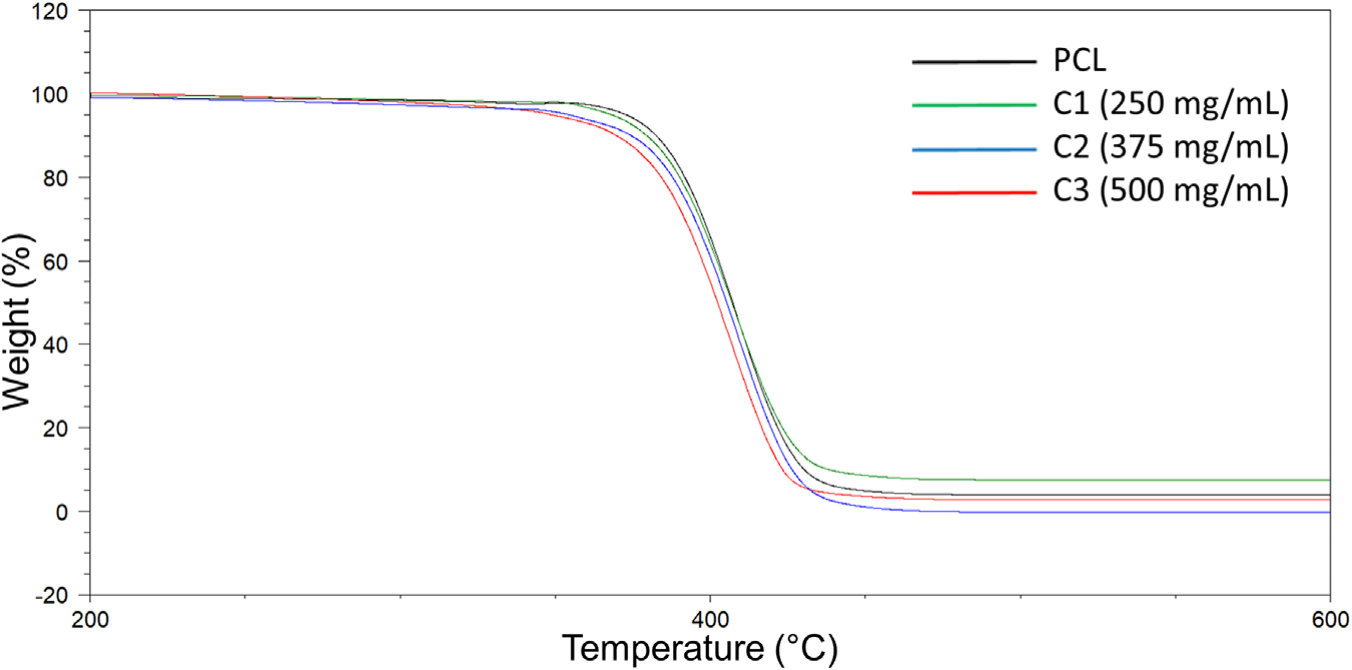
TGA thermograms of PCL (15%), C1: 15% PCL+250 mg/mL cinnamon, C2: 15% PCL+375 mg/mL cinnamon, and C3: 15% PCL+500 mg/mL cinnamon gyrospun polymeric fibers between 200°C and 600°C

Thermal decomposition temperatures and weight loss % data obtained by the thermal gravimetric analysis (TGA) (Figure 2) shows that the blending of PCL with cinnamon extract causes a slight reduction in the thermal stability of the fibers. TGA results were collected between 200 and 600°C. The temperatures of the PCL, C1, C2, and C3 weight loss at *T_X%_* are given in detail in Table 1 for *T*_5%_, *T*_10%_, *T*_15%_, *T*_20%_, *T*_50%_, *T*_75%_, *T*_90%_, and *T*_100_ *_%_* (°C), respectively. Thermal decomposition temperature values were decreased due to the presence of cinnamon and increased content ratio. A downward trend was observed at the all weight loss ratios (*T_X%_*).

**TABLE 1:** Temperature values of virgin PCL, C1, C2, and C3 gyrospun polymeric fiber materials in terms of weight loss %

Fiber sample	*T*_5%_ (°C)	*T*_10%_ (°C)	*T*_15%_ (°C)	*T*_20%_ (°C)	*T*_50%_ (°C)	*T*_75%_ (°C)	*T*_90%_ (°C)	*T*_100%_ (°C)
**PCL**	372.7	382.5	387.8	391.8	407.2	418.8	430.3	479.9
**C1**	368.1	379.5	385.8	390.2	406.9	419.8	438.0	480.0
**C2**	354.4	374.5	382.7	387.8	405.3	416.9	426.4	474.6
**C3**	348.7	369.8	378.6	384.2	402.4	414.0	423.3	478.7

### 2.3 Cytotoxicity against fibroblast cells of the fibrous samples

**FIGURE 3:**
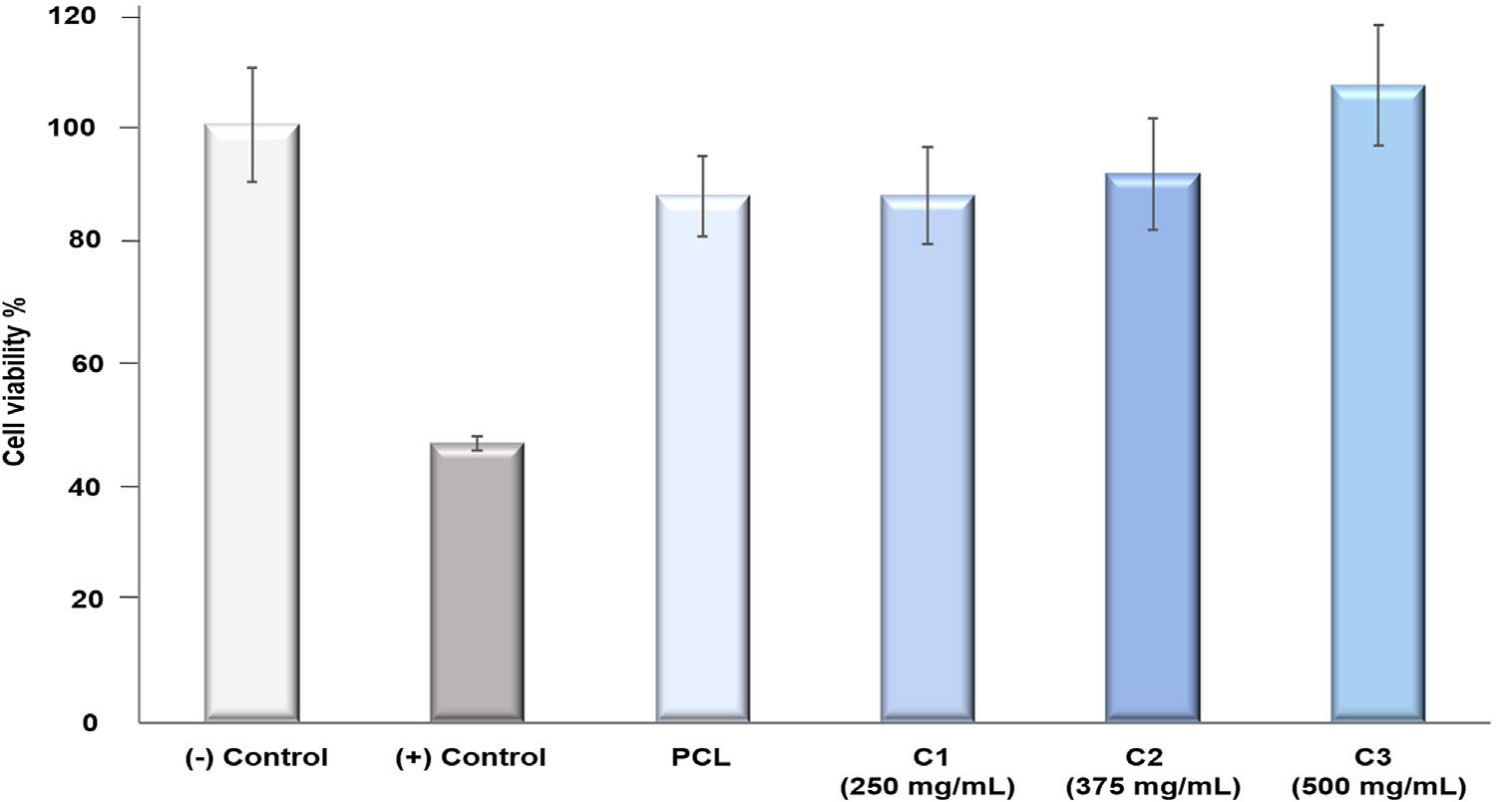
Cell cytotoxicity test results of PCL and cinnamon‐extract containing C1, C2, and C3 gyrospun polymeric fibers with the different concentrations compared to DMEM negative control and 10% DMSO containing DMEM positive control. C1: 15% PCL+250 mg/mL cinnamon, C2: 15% PCL+375 mg/mL cinnamon, and C3: 15% PCL+500 mg/mL cinnamon

**FIGURE 4:**
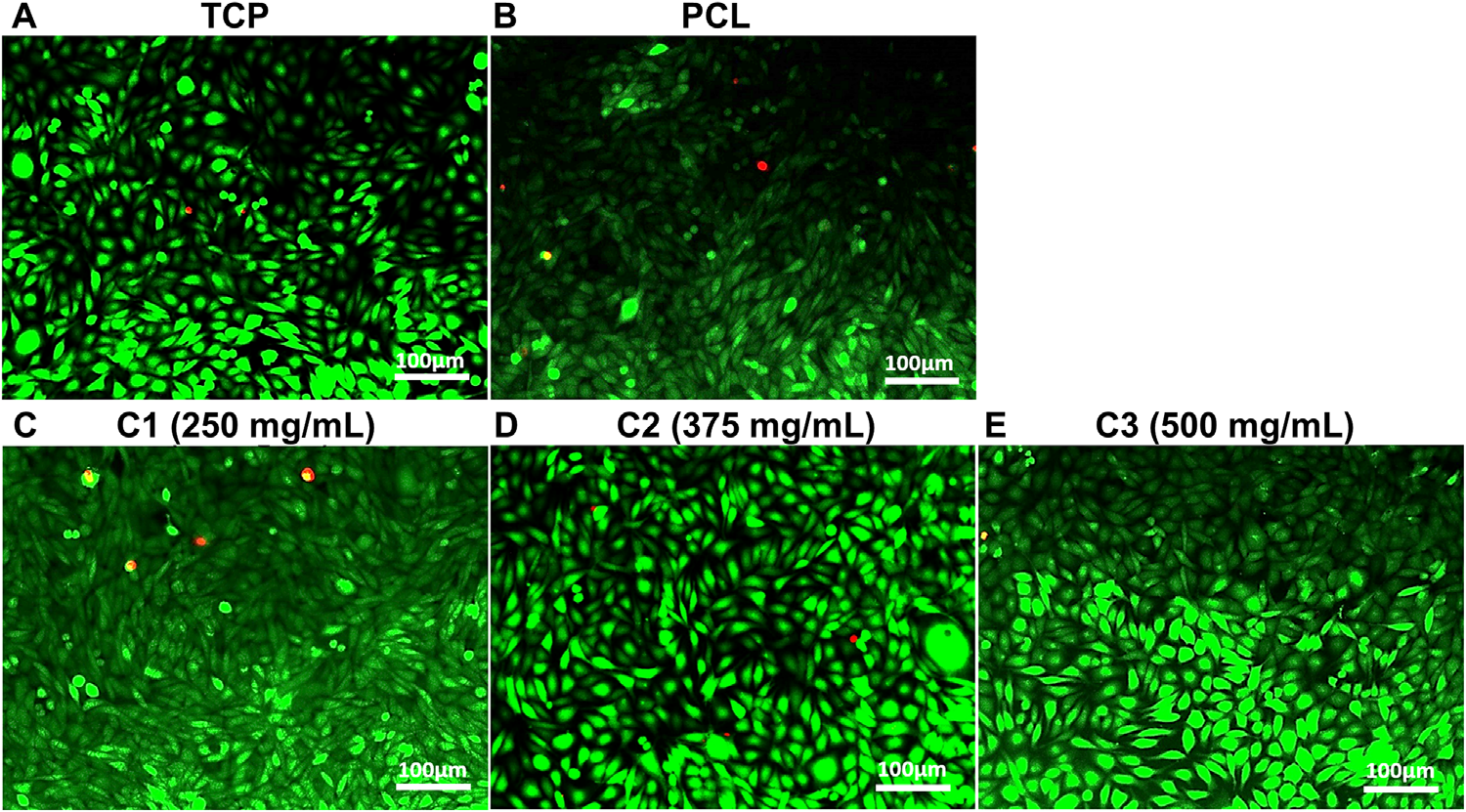
Fluorescence microscope images showing the live/dead staining for: (A) TCP, (B) virgin PCL fibers, (C) C1 fibers, (D) C2 fibers, and (E) C3 fibers. These show the live/dead stain images for the fibrous samples and the PCL fibers without cinnamon extract. Tissue culture plate (TCP) is used as negative control during the experiment. Similar cell viability was observed at each test and control groups. It is evident from the vast majority of live cells that all samples were able to sustain a high degree of cell viability or that the inclusion of cinnamon extract to the biocompatible PCL does not adversely affect its toxicity to cells

Cytotoxicity tests (Figure 3) where carried out with the L929 mouse fibroblast cell line for all the fibrous samples and the two controls. Cell cytotoxicity is an important consideration when dealing with biomaterials and any polymeric structure, which comes into contact with body fluid. Cytotoxicity assays performed in this work therefore show the cell viability of and the cytotoxic effects of cinnamon‐extracted PCL fibers at the four tested concentrations (Virgin PCL, C1, C2, and C3 fibers). A negative control Tissue Culture Plate (TCP) was used and a positive control, which included 10% dimethyl sulfoxide (DMSO). Compared to the negative control, a large reduction in cell viability would indicate that the hosts cells may have their cell membrane integrity compromised if the cell viability fell below the acceptable threshold of 70%. We can see that all fiber samples maintained significantly higher viability than the cytotoxic threshold. Additionally, live/dead staining test (Figure 4) was performed for each sample group and the results correlated with the indirect cytotoxicity test results. All the sample groups C1, C2, and C3 exhibited high cell viability with very rare dead cells, which were shown with red dots on florescence images.

### 2.4 Antibacterial activity of the fibrous samples against *E. coli, S. aureus, MRSA*, and *E. faecalis*

**FIGURE 5:**
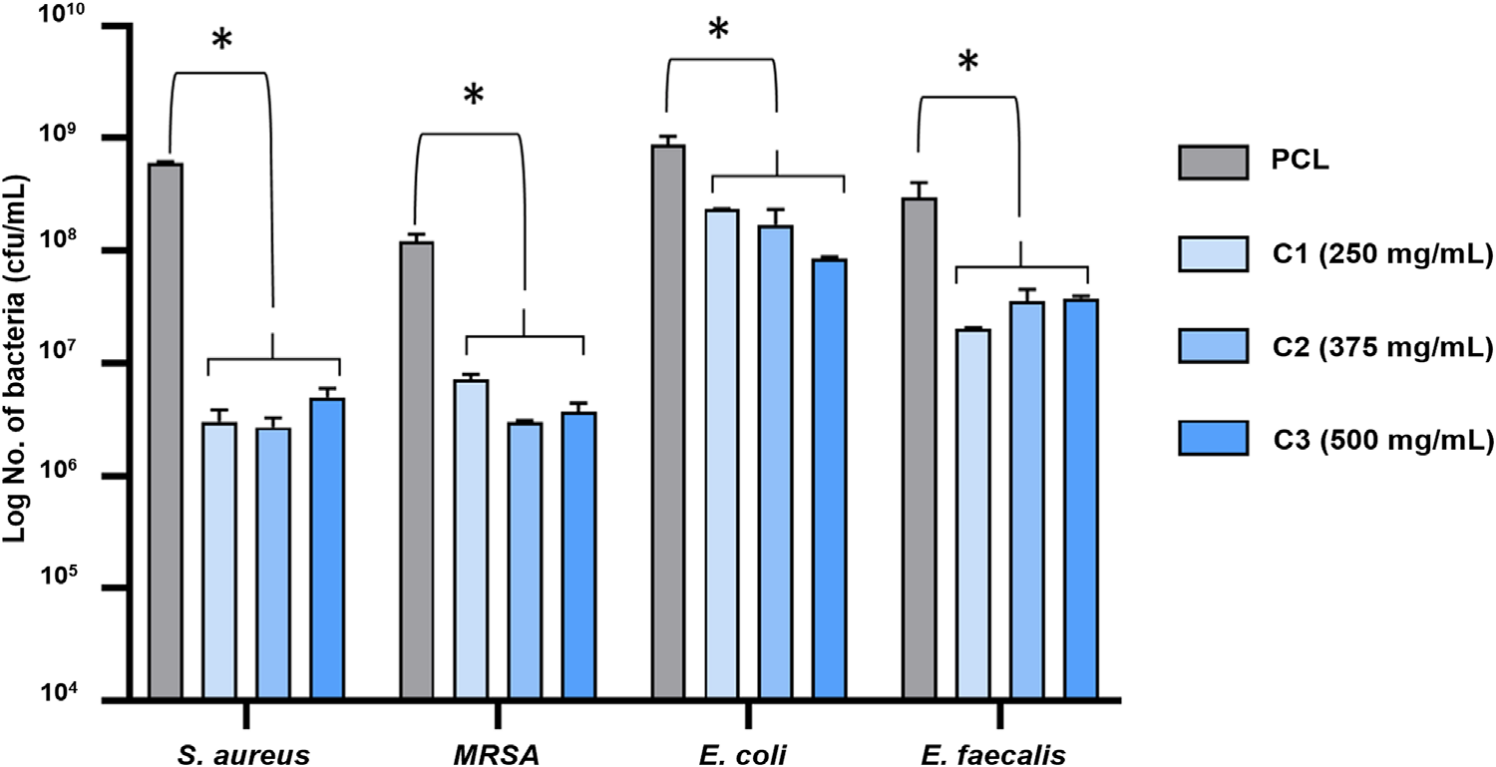
Antibacterial test results for control (virgin 15% PCL), C1: 15% PCL+250 mg/mL cinnamon, C2: 15% PCL+375 mg/mL cinnamon, C3: 15% PCL+500 mg/mL cinnamon gyrospun polymeric fibers against *S. aureus*, MRSA, *E. coli*, and *E. faecalis* bacteria species (*p < 0.05)

**FIGURE 6:**
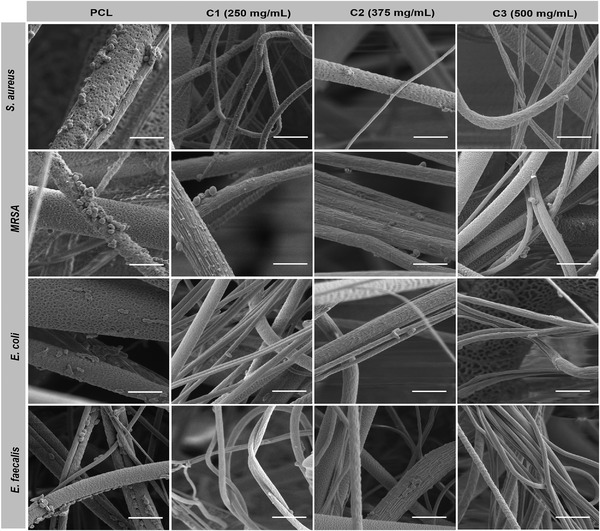
SEM pictures of antibacterial test results for control (virgin 15% PCL); C1: 15% PCL + 250 mg/mL cinnamon, C2: 15% PCL+375 mg/mL cinnamon, and C3: 15% PCL + 500 mg/mL cinnamon gyrospun polymeric fibers against *S. aureus*, *MRSA*, *E. coli*, and *E. faecalis* species. For each micrograph, scale bar corresponds to 5 μm

Antibacterial tests were performed against *E. coli, S. aureus, MRSA*, and *E. faecalis* strains over a 24‐hour incubation period for the PCL control and cinnamon extracted fiber samples. The results of the antibacterial tests are presented in Figure 5, where significant differences are denoted with an asterisk (*p < 0.05). Compared to the virgin PCL samples, all tested fibers with cinnamon loading showed a significantly higher antibacterial activity in the way of reducing the bacterial cell count. The antibacterial test results were also investigated under high magnification using SEM, these images are given in Figure 6. The SEM micrographs support the results obtained from the antibacterial test results. PCL alone, with no native antibacterial capability was compared to the active cinnamon containing fibers (C1: 250 mg/mL, C2: 375 mg/mL, and C3: 500 mg/mL). The presence of bacterial colonies on the surface of the PCL fibers indicates that the surface of the material does not actively prevent bacterial formation and as such, bacteria are able to sustain a population onto them.

## 3 DISCUSSION

The average fiber diameter for these PCL based fibers was 2.8 ± 3.7 μm for the C1 sample, 2.3 ± 2.2 μm for the C2 sample, and 1.7 ± 1.4 μm for the C3 sample. There is a slight decrease in fiber diameter observed when increasing the cinnamon extract concentration perhaps due to the reduction of polymer chains unfolding during the dissolution process as there is an increased presence of molecules provided by the increase in cinnamon concentration.^44^ The finer fiber diameter allows for an increased surface area where the antimicrobial activity can be enhanced by an increase in small‐molecule release rates.^45^ Furthermore, differences in fiber diameter can be useful in the treatment and prevention of infections, especially related to orthopedic trauma surgery where changes in diameter can affect the phenotypical responses from osteoblast and fibroblast cells.^46^ It has been shown that the tailoring of fiber diameter of these fibrous scaffolds can be done with ease with our current manufacturing process used here and allows for a high degree of customizability for each intended application.^47^, ^48^, ^49^ We also observe that the fiber diameter uniformity also increases marginally when increasing the extract concentration. Lower uniformity in size distribution can be advantageous in some applications where a randomized mesh can increase particulate filtration, such as in masks, and high uniformity is advantageous in tissue engineering applications.^50^, ^51^

The starting decomposition temperature reduced with increasing concentration of cinnamon extract. The C1, C2, and C3 blends lost 5% of their weights at a temperature of 368.1°C, 354.4°C, and 348.7°C, respectively, whereas PCL loses 5% of its weight at 372.7°C. The temperatures of the PCL, C1, C2, and C3 weight loss at *T_X%_* are given in detail in Table 1 for *T*_5%_, *T*_10%_, *T*_15%_, *T*_20%_, *T*_50%_, *T*_75%_, *T*_90%_, and *T*_100%_ (°C), respectively. The results presented here show discernible difference in the thermogravimetric values for the different samples, suggesting that the increase in cinnamon concentration may have an effect on the adsorption capability of the samples.^52^, ^53^

Cytotoxicity tests (Figure 3) showed that there is no significant toxic effect for all the fiber samples tested (*p* < 0.05). For both the virgin PCL fibers and the three cinnamon‐extract containing fiber samples, that cell viability remained high. Virgin PCL fibers showed a cell viability of 88.1 ± 6.7% compared to the negative control, which retained a 100% cell viability. The cinnamon‐extract containing fibers demonstrated cell viability as follows: C1 samples had a viability of 88.1 ± 8.1%, C2 samples had a viability of 91.8 ± 9.2% and C3 samples had 106.6 ± 10% cell viability, which is considered to indicate a high degree of cytocompatibility, especially when compared to the results of the positive control (46.7 ± 1.1% cell viability). According to the ISO10993‐5 standard, the threshold limit for accepted cell viability is 70%, where falling under this threshold would be considered as cytotoxic. Therefore, all the tested samples can thus be deemed as having acceptable levels of cytotoxicity, which should not pose as a concern for bandage and other healthcare materials. As a result, cinnamon blended PCL fibers demonstrate promising potential for the use of gyrospun fibers as biomaterials with a novel combination of natural materials.

We can see that all the tested samples illustrate high cell viability, which is evident from the low number of dead cells from (Figure 4).^54^ Direct live/dead staining test results support these results obtained from the indirect (3‐(4,5‐dimethylthiazol‐2‐yl)‐2,5‐diphenyltetrazolium bromide (MTT assay) tests. Samples C1, C2, and C3 all demonstrated high cell proliferation/viability similar to the control group and the virgin PCL sample. PCL is widely used as a biomaterial in tissue engineering as it is FDA (Food and Drug Administration) approved, low cost, biocompatible, biodegradable, and easy to manufacture.^55^ Cinnamon is also a well‐known natural anti‐inflammatory agent but is let down with its low‐solubility and potential allergic reactions for some users.^56^ Salehi and coworkers also indicated that cinnamon is very effective for wound healing applications both *in vitro* and *in vivo*.^57^ The cytotoxicity tests here alleviate some concern in the toxicity of cinnamon‐extracted materials, which have been produced as dry fibers.

The results from the antibacterial tests (Figure 5) showed that C1, C2, and C3 samples decreased the bacterial cell numbers by up to 2 orders of magnitude (log 2 / 100 times). The sample groups C1, C2, and C3 showed statistically significant decreases (*p < 0.05) in the bacterial cell viability in contrast to the PCL control group for *E. coli* (Gram negative), *S. aureus* (Gram positive)*, MRSA* (Gram positive), and *E. faecalis (*Gram positive). The antibacterial test results showed that the bacterial cell numbers on the PCL control groups were 6.1 × 10^8^, 1.2 × 10^8^, 8.7 × 10^8^, and 3 × 10^8^ cfu/mL for *S. aureus*, MRSA, *E. coli*, and *E. faecalis*, respectively. The cinnamon extracted bandage like fibrous sample groups exhibited statistically significant decreases as follow: C1: 3 × 10^6^ cfu/mL, C2: 2.7 × 10^6^ cfu/mL, C3: 5 × 10^6^ cfu/mL for *S. aureus*, C1: 7.2 × 10^6^ cfu/mL, C2: 3.1 × 10^6^ cfu/mL, C3: 3.7 × 10^6^ cfu/mL for *MRSA*, C1: 2 × 10^8^ cfu/mL, C2: 1.7 × 10^8^ cfu/mL, C3: 9 × 10^7^ cfu/mL for *E. coli*, and C1: 2 × 10^7^ cfu/mL, C2: 3.6 × 10^7^ cfu/mL, C3: 3.8 × 10^7^ cfu/mL for *E. faecalis*. It has been shown that all the sample groups exhibited statistically significant reduction in the bacterial cell viability compared to the PCL control group. On the other hand, there is no observable significant difference between C1, C2, and C3 sample groups against any of the bacterial species, this could mean that even at the lowest concentration, there is enough active ingredient to cause an antibacterial effect.

Figure 6 shows the PCL control group against S*. aureus, MRSA, E. coli*, and *E. faecalis* strains, respectively. In these micrographs, bacterial species adhere to the fiber structures with higher numbers compared to the C1, C2, and C3 sample groups for each tested bacterial species. Additionally, in the control groups, the adhered bacterial cells managed to colonize but the cells on C1, C2, and C3 sample groups showed random and rare colonies with fewer cell numbers than shown in Figure 6.

The results showed that cinnamon‐extract containing PCL fibers successfully exhibited antibacterial activity for both Gram‐negative and Gram‐positive species. Additionally, *E. coli, a* Gram‐negative species, showed statistically significant decrease in the viable cell number but was not as much when compared to the Gram‐positive species. This result can be explained by the differences between the cell membranes of the bacterial species. This is because the bacterial membrane of Gram‐negative species are thicker than the positive counterparts as they have the lipopolysaccharide peptidoglycan cell membrane.^58^ The antibacterial mechanism of cinnamon is via disruption of bacterial cell wall and subsequent penetration through the cell wall to demolish the cytoplasmic membrane.^59^, ^60^ The antibacterial activity of cinnamon has long been studied in literature, especially in relation to food and cosmetic applications.^61^, ^62^ Cinnamon‐extracted and other spice‐extraction methods have not been translated into the production of polymeric biodegradable bandages before. Synthetic compounds can have detrimental environmental impacts in the long term such as the large necessity for crude oil, toxic run‐off and nondegradable release mechanisms. The work here validates the viable means of producing antibacterial biomaterials that have a high antimicrobial efficacy whilst remaining within safety limits and offering to be more environmentally responsible and cytocompatible than synthetic approaches.

From the results presented here, we see that cinnamon‐extract containing polymeric fibrous biomaterials can deliver significant antibacterial properties against *S. aureus*, MRSA, *E. coli*, and *E. faecalis* species. These species frequently contribute to and cause common hospital acquired infections, which increases in prevalence in open wound environments, surgical operations, hernia, and infected interocutaneous fistula. The spice‐extract containing bandage‐like materials do not display significant cytotoxicity and can thus be safely used as bandage materials to cover wounds and prevent infections, as a filter material for masks or even as a biomaterial. The production method and the tailorable nature of the production methods allow for precise control in manufacturing, leading to fibrous scaffold materials, which can sustain the necessary requirements of a good antibacterial biomaterial environment. Thinner diameter fibers can be produced to increase the available surface area for bioactivity and the release of beneficial pharmaceutical or medicinal ingredients. The ease of use, value, and rapid manufacturing parameters will be the key to outstanding potential. The advantages of these natural cinnamon‐extract containing bandages are vast and pose as a contender to the future of bioactive biomaterials with a fiber‐based architecture.

## 4 METHODS AND MATERIALS

### 4.1 Fiber preparation

Cinnamon was extracted by solvent extraction from pure ground cinnamon (*cassia*) powder (JustIngredients, UK). Chloroform (CAS Number: 67‐66‐3, Sigma Aldrich, UK) was used to extract and create three varying concentrations of cinnamon‐extracted polymer solutions. Cinnamon extracts of 250, 375, and 500 mg/mL (C1, C2, and C3, respectively) were made and PCL polymer pellets (Sigma Aldrich, UK) were mixed into the cinnamon solutions and mechanically stirred for 24 hours to form a homogenous polymer solution.

The PCL polymer solutions were then loaded into a pressurized gyration device consisting of a compact aluminum (35 mm × 60 mm) cylindrical vessel with 24 circular perforations (area = 0.5 mm^2^), connected to a gas inlet and a high speed motor to produce fibrous meshes.^63^, ^64^ Four milliliters of the polymer solution were spun by pressurized gyration for a duration of 20 seconds, which resulted in the production of bandage‐like polymeric meshes. All production occurred at ambient conditions (24°C and 42% relative humidity), and the gyratory speed was set at 36,000 rpm with 0.1 MPa pressure.

### 4.2 Fiber characterization

The fibrous samples were examined by SEM. Samples were gold sputter‐coated for 90 seconds (Q150R ES, Quorum Technologies) and were subsequently imaged under high magnification (Hitachi S‐3400n). The resulting micrographs were then analysed using Image J software, 100 fiber strands were measured at random and the mean diameter and its accompanying distribution was plotted using Origin pro software.

The chemical structures of the cinnamon‐extract containing bandage‐like fiber constructs were characterized by thermal gravimetric analysis (TGA) (TA Instruments Q600‐SDT, USA) to investigate and compare the thermal characteristics of PCL and cinnamon extract containing‐PCL fiber samples.

### 4.3 Cytotoxicity of cinnamon‐extract containing fiber constructs

Cytocompatibility tests were performed for PCL and PCL‐cinnamon gyrospun fibers according to the ISO10993‐5 standard “Biological evaluation of medical devices‐Part 5. Tests for *in vitro* cytotoxicity: Indirect MTT cytotoxicity test” regulation.^65^ L929 (ATCC‐NCTC clone 929:CCL1) mouse fibroblast cell line was used in this study. Incubation conditions were kept constant at 37°C, >90 % humidity, and at 5% atmospheric CO_2_. Dulbecco's modified Eagle's medium (DMEM) (90% (v/v)) and fetal bovine serum (FBS) (10% (v/v)) were used as the cell culture medium in the presence of 0.1% penicillin‐streptomycin. Fiber samples were incubated in the cell culture medium for 3 days with 0.2 g/mL fibers/cell culture medium ratio in the incubator at 37°C. L929 cells were seeded into a 96‐well plate with a cell count of 1 × 10^4^ per well. Extracted cell medium solutions interacted with the cell line in the well plate for 24 hours. 10% DMSO containing cell medium was used as a positive control and DMEM‐FBS medium was used as a negative control. At the end of the study, L929 cells were incubated with 10% (4,5‐Dimethylthiazol‐2‐yl)‐2,5‐diphenyltetrazolium bromide (MTT assay) solution for 3.5 h at 37°C, MTT crystals were dissolved then by DMSO. Cell viability percentages were determined by the ELISA micro plate reader at an absorbance of 570 nm.

Additionally, direct cell‐material interactions were investigated by LIVE/DEAD™ viability/cytotoxicity tests. The same cell culture conditions and number of cells as the MTT test were used. 1 × 10^4^ numbers of L929 cells were seeded from each well onto the fiber samples directly. After a 3‐day incubation period, the LIVE/DEAD™ Viability/Cytotoxicity staining kit was used to obtain live and dead cell fluorescent images of samples. Ethidium homodimer‐1 (EtdH‐1) (4 μM, red) and Calcein AM (2 μM, green) fluorescent dyes were mixed in phosphate buffer saline (PBS) and incubated with the samples for 45 minutes. At the end of the incubation period, samples were examined by fluorescent microscopy (Leica Microsystems, Germany). Fluorescence microscope images indicated red for dead cells and green for live cells, images were then overlaid (ImageJ 1.52a, USA).

### 4.4 Antibacterial activity of cinnamon‐extract containing fiber constructs

The antibacterial activity of cinnamon‐extract containing bandage‐like fibers was investigated by a 24‐hour bacterial adherence/biofilm formation assay. *Escherichia coli* (*E. coli* ATCC 25922), *Staphylococcus aureus* (*S. aureus* ATCC 29213), *Methicillin* Resistant *Staphylococcus aureus* (*MRSA* ATCC 43300), and *Enterococcus faecalis* (*E. faecalis* ATCC 29212) strains were used. Tested bacterial isolates were dissolved from stocks kept at –80°C in glycerol medium and sub‐cultured twice prior to use. Each of the strains were adjusted to approximately 1 × 10^6^ colony forming unit/mL (CFU/mL) as a final inoculum suspension concentration. One square centimeter of the samples was placed into a 48‐well plate. One milliliter of bacterial suspension was added to the samples in each well. Well plates were incubated at 37°C for 24 hours in a shaking incubator. After 24 hours, samples were rinsed with sterile PBS, moved into a new sterile well plate and sonicated (Branson Sonifier SFX250, USA) in 1 mL sterile PBS medium (15 minutes). Sample solutions were serially diluted and placed into petri dishes. Tryptic soy agar (TSA) medium was poured onto the dishes and stirred. After 24‐hour incubation, each Petri dish was counted for dilutions and biofilm formation was investigated.

For the SEM analysis (SEM, GAIA 3; Tescan, Brno, Czech Republic), 24 hours after incubation with bacterial suspensions, samples were rinsed with PBS and fixed. Samples were kept in glutaraldehyde (G.A) solution (2.5% v/v, GA/PBS) for 15 minutes. Samples were washed with serial alcohol dilutions for 15‐minute time intervals (25%, 50%, 70%, 80%, 90%, 95%, and 100% ethanol) and hexamethyldisilazane (HMDS) was added to the top of completely dry samples.^66^ Prior to SEM analysis, samples were coated in a thin layer of gold via sputter coating (Edwards, UK).

### 4.5 Statistical analysis

Antibacterial activity data sets were analyzed according to one‐way ANOVA test. Tukey's test was used to perform statistical analysis and a *p* value of < 0.05 was considered as statistically significant.

## CONFLICTS OF INTEREST

Author Mohan Edirisinghe is the Associate Editor of MedComm. Author Mohan Edirisinghe was not involved in the journal's review of, or decisions related to, this manuscript.

## ETHICS APPROVAL

Ethical committee approval is not required. The cell and bacterial resources are used as cell lines and bacteria strains in the cell/bacteria studies performed in the related study.

## AUTHOR CONTRIBUTIONS

JA and ME conceived the initial concept. JA and MG designed the study. JA, MG, CB, DK, and KU performed the experiments. JA and MG wrote the paper.

## Data Availability

The raw/processed data required cannot be shared at this time as the data also forms part of an ongoing study.
